# Two Cases of Stenosis Uteri with Symptoms of Locomotor Ataxy

**Published:** 1883-04

**Authors:** O. Stroinski


### ARTICLE VIII.

### TWO CASES OF STENOSIS UTERI WITH SYMPTOMS OF LOCOMOTOR ATAXY. By O. STROINSKI, M.D.

I. Mrs. M. N., a rather strong woman, fifty-two years of age, was carried to my office with the assistance of two persons. She had always been a healthy person but had borne no children. Three years ago, and one year after the menopause, she noticed rheumatoid pains in the right lower limb, which also attacked the other lower extremity. After a while there was added to that a sensation of cold and numbness is the same regions; and she often could not distinguish which was the right and which was the left leg. Then she had a sensation of whirling around when awakening in the morning and when leaving the bed she would be unable to stand upon her feet for a quarter of an hour. Urinary trouble also set in and the sphincter ani gave away temporarily. Later, the pains in the lower extremities increased and they attacked the forearms and the hands. Pains in the groins appeared and the walk was gliding. All the symptoms of locomotor ataxy had now gradually developed, but vision was unimpaired and ophthalmoscopic examination showed the retina to be perfectly normal. The galvanic current acted well on the several affected groups of muscles, and Westphal's knee phenomenon could not be elicited. The latter symptoms, in connection with the healthy and stout appearance of the woman induced me to examine the genital tract. The ovaries were not palpable and the parts adjacent to the uterus showed no irregularities.. The cervix uteri was completely occluded by agglutination of the walls of the cervix and it was impossible to enter the cavity with the uterine sound even by increased pressure. I therefore perforated the cervix with a small trocar and after dilating the same with laminaria tents, introduced a common intra-uterine pessary. After six weeks the phenomena of locomotor disturbance began to disapper and micturition was normal. Afterward it was necessary to apply the iutra-uterine stem every three or four months, the labia of the cervix inclining to agglutinate again.

II. I was called to see Mrs. Anna W., fifty-three years of age, who had been unable to leave her bed for the last three months. Four years ago and about one year and a half after the menopause, she noticed the same pains as above described, and the course of the disease was similar to that of Mrs. N. But here the sphincter ani was more involved, and she lost all power of motion. There was also constant constipation caused by partial paralysis of the bowels; and for the last four months she had to make injections with large quanties of water to induce defecation. Anæsthesia was complete in the lower extremities and the galvanic current acted but slowly on the muscles, especially on the gluteus maximus and biceps. The pulse was 50, and the action of the heart retarded. The sphincter ani could be passed by two fingers without producing any pain, and there was no difficulty in introducing a large sized bougie into the bladder. The cervix was closed as in the first case, and it was treated in the same manner. Improvement was more rapid in this case and the woman was able to do her house work after two months.

These cases are very rare and they are mostly diagnosed as locomotor ataxy, but they are known to exist by every gynæcologist. The pathological condition of these cases has never been examined and it has been hitherto impossible to decide as to the symptoms produced, especially in those cases where the uterus is no longer irritated by the monthly ripening of the ova. It is probable that pressure on the uterine nerves caused by the agglutination of the uterine walls acts reciprocally, on the nervous system of the abdominal part of the body and on that of the spinal cord.

---

### THE BACILLUS OF GLANDERS.

The President of the Imperial Board of Health, Dr. Struck, at Berlin, publishes the results of experiments made on glanders. The material was taken from nodules in the diseased mucous membrane of horses which had died from the disease. Sections of these nodules were colored with a saturated solution of methylene-blue, washed with diluted acetic acid, bathed in alcohol, and then imbedded in cedar oil. There were small bacilli, which were cultivated in horse blood. The products of these cultures, injected hypodermically into several parts of mice, rabbits and guinea pigs, produced a disease which showed all the symptoms of glanders, such as swelling of the testicles and ovaries, with ulcerative processes in the nasal cavities, and general infection, with final death. Injections with material derived from these animals had also always produced the same results.—*Wiener Med. Wochenschrift.*

